# Using *Drosophila* to discover mechanisms underlying type 2 diabetes

**DOI:** 10.1242/dmm.023887

**Published:** 2016-04-01

**Authors:** Ronald W. Alfa, Seung K. Kim

**Affiliations:** 1 Department of Developmental Biology, Stanford University School of Medicine, Stanford, CA 94305, USA; 2 Neuroscience Program, Stanford University School of Medicine, Stanford, CA 94305, USA; 3 Howard Hughes Medical Institute, Stanford University School of Medicine, Stanford, CA 94305, USA; 4 Department of Medicine (Oncology), Stanford University School of Medicine, Stanford, CA 94305, USA

**Keywords:** Diabetes, *Drosophila*, Insulin resistance, Insulin-like peptides

## Abstract

Mechanisms of glucose homeostasis are remarkably well conserved between the fruit fly *Drosophila melanogaster* and mammals. From the initial characterization of insulin signaling in the fly came the identification of downstream metabolic pathways for nutrient storage and utilization. Defects in these pathways lead to phenotypes that are analogous to diabetic states in mammals. These discoveries have stimulated interest in leveraging the fly to better understand the genetics of type 2 diabetes mellitus in humans. Type 2 diabetes results from insulin insufficiency in the context of ongoing insulin resistance. Although genetic susceptibility is thought to govern the propensity of individuals to develop type 2 diabetes mellitus under appropriate environmental conditions, many of the human genes associated with the disease in genome-wide association studies have not been functionally studied. Recent advances in the phenotyping of metabolic defects have positioned *Drosophila* as an excellent model for the functional characterization of large numbers of genes associated with type 2 diabetes mellitus. Here, we examine results from studies modeling metabolic disease in the fruit fly and compare findings to proposed mechanisms for diabetic phenotypes in mammals. We provide a systematic framework for assessing the contribution of gene candidates to insulin-secretion or insulin-resistance pathways relevant to diabetes pathogenesis.

## Abstract (precis)

***Drosophila* Collection:** Studies in *Drosophila* hold the potential to transform our understanding of the genetics of diabetes. We review important findings and provide a framework for diabetes gene discovery in the fly.

## Introduction

At a global prevalence exceeding 9% of the human population, type 2 diabetes mellitus (T2D) is frequently cited as a global pandemic (World Health Organization Publications, 2014). Although the undeniable connection between T2D and obesity in Western societies has fueled much research into behavioral and environmental causes, it has long been known that only a subset of obese individuals progress to diabetes and that this susceptibility is heavily influenced by genetics (Bouret et al., 2015; Eckel et al., 2011; Kahn et al., 2014). Thus, understanding the mechanisms underlying differential susceptibilities among individuals and populations provides an opportunity to identify new molecular markers and targets for therapeutic intervention. Genome-wide association studies (GWAS; Box 1) have enabled progress toward this goal by identifying over 90 loci associated with diabetic phenotypes (Dimas et al., 2014; Frayling and Hattersley, 2014; Renström et al., 2009). Nonetheless, major challenges remain in translating GWAS associations into mechanistic and clinically translatable insights (McCarthy et al., 2008). As discovery of disease-associated single-nucleotide polymorphisms (SNPs) continues, these SNPs first need to be causally associated with individual genes. Once gene candidates are identified, the gold-standard for characterizing the molecular mechanisms of disease alleles and the role of individual genes in metabolic disease is experimental interrogation in model organisms (McCarthy et al., 2008). This task can present a formidable challenge considering that SNPs might cause gain of function, loss of function or reflect tissue-specific effects. *Drosophila melanogaster* is a highly suitable system to model defects in these pathways both because mechanisms of glucose homeostasis are conserved between flies and humans, and the fruit fly allows for substantial ease of experimental and genetic manipulation in comparison to rodent models.

**Box 1. Glossary**

**Genome-wide association study (GWAS):** study that examines the association between large numbers of genetic variants [e.g. single-nucleotide polymorphisms (SNPs)] and a particular disease or disease phenotypes. GWAS uses statistical methods to identify variants that occur more frequently in individuals with a disease or disease trait. Associated variants can be localized to coding or non-coding regions of the genome.

**Large dense-core vesicles (LDCVs):** subcellular organelles involved in the trafficking, processing, storage and secretion of peptide and neuropeptide hormones.

**Stimulus-secretion coupling:** the process and mechanisms by which an extracellular glucose stimulus is transduced into membrane excitability and insulin secretion from pancreatic β-cells. The process begins with the cellular uptake of glucose through glucose transporters and ends with the calcium-dependent vesicle fusion and release of insulin from secretory vesicles.

**Pre-propeptides:** immature peptide precursors that undergo post-translational processing to yield bioactive peptides. Precursors undergo removal of the signal peptide in the ER to yield propeptides (e.g. pro-insulin). Bioactive peptides are produced through further processing of propeptides by prohormone convertases in secretory vesicles, including: cleavage and removal of fragments, disulfide-bond formation and additional biochemical modification of amino acid residues.

**Membrane depolarization:** neurons and other electrically excitable cells maintain a net charge separation across their membrane (intracellular more negative than extracellular) through the selective distribution of anions and cations. Depolarization occurs when changes in ion channel permeability permit redistribution of ions (e.g. influx of cations Na^+^, Ca^2+^) across the cell membrane, resulting in an increase in positive charge within the cell.

The pathophysiological hallmarks of T2D in mammals are an impaired response of peripheral tissues to insulin (insulin resistance) and impaired insulin secretion from pancreatic β-cells (Kahn et al., 2014; Weyer et al., 1999). Early on in T2D development, insulin resistance leads to compensatory elevation of insulin secretion, which counteracts the decrease in tissue sensitivity and maintains normal blood glucose levels by stimulating uptake by tissues such as adipose and liver (Kahn et al., 2014; Kasuga, 2006). Elevated circulating glucose levels (hyperglycemia) and T2D result from a mismatch of insulin demand and activity, for example β-cell dysfunction in the face of insulin resistance (Kahn et al., 2014). Nonetheless, T2D occurs across a spectrum of insulin resistance, and GWAS candidates have been found to associate independently with either insulin sensitivity or insulin secretion (Dimas et al., 2014; Zhao et al., 2010). This suggests that expression of diabetic phenotypes might be due to independent susceptibilities in each of these domains, with diverse combinations of genetic susceptibilities contributing to disease within a given population.

**Fig. 1.**
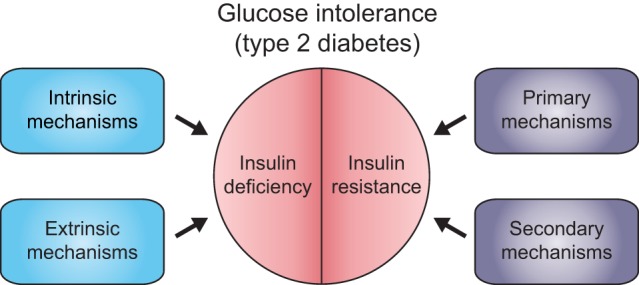
**Genetic pathways to glucose intolerance.** The diagram depicts a simplified framework for organizing the molecular mechanisms underlying diabetic phenotypes in model organisms and humans. Glucose intolerance (hyperglycemia) and type 2 diabetic phenotypes result from the combination of insulin resistance and functional insulin deficiency due to inadequate compensation, i.e. inadequate upregulation of insulin output. Insulin resistance results from primary defects (primary mechanisms) in insulin/IGF-like signaling (IIS) or through secondary mechanisms that prevent insulin from binding to its receptor or disrupt effectors downstream of IIS. Conversely, mutations that cause insulin deficiency phenotypes affect genes involved in the secretion of insulins (intrinsic mechanisms) or the non-autonomous modulation of insulin production or secretion (extrinsic mechanisms).

A simplified framework for conceptualizing the physiological mechanisms giving rise to glucose intolerance in humans and model organisms is outlined in Fig. 1. Insulin production and secretion (collectively referred to as insulin output) from endocrine cells are modulated by cell-intrinsic and cell-extrinsic mechanisms. Intrinsic mechanisms include cellular processes that regulate insulin transcription, translation or secretion. Extrinsic mechanisms include neuro-humoral signals that modulate the steady state of insulin production or secretion. Defects in any of these pathways give rise to absolute or relative insulin deficiency. In contrast, insulin resistance refers to the decreased response of peripheral tissues to insulin signaling. To organize discussion of this topic, we differentiate between *primary* mechanisms of insulin resistance – due to impaired insulin-to-insulin-receptor signaling – and *secondary* mechanisms of insulin resistance, such as impaired glucose uptake or inappropriately elevated glucose production. Hyperglycemia due to insulin deficiency alone, such as with endocrine cell destruction in type 1 diabetes (T1D), reflects decreased insulin output with normal or increased sensitivity, whereas hyperglycemia due to insulin-resistant states (such as in T2D) can be accompanied by either increased insulin output (because of ‘compensation’ by cells that produce insulin) or decreased insulin output (failed compensation).

Here, we review relevant findings from studies modeling T2D and glucose homeostasis in *Drosophila* (see Table 1). We begin with a brief introduction to glucose homeostasis in the fly. In the sections that follow, we review the molecular mechanisms governing insulin output and insulin sensitivity, and illustrate how the framework described above (Fig. 1) can be used to characterize the function of additional diabetes gene candidates, including those nominated by human GWAS studies. These studies demonstrate how the fruit fly can be leveraged to accelerate research into the molecular mechanisms underlying T2D.

**Table 1.**
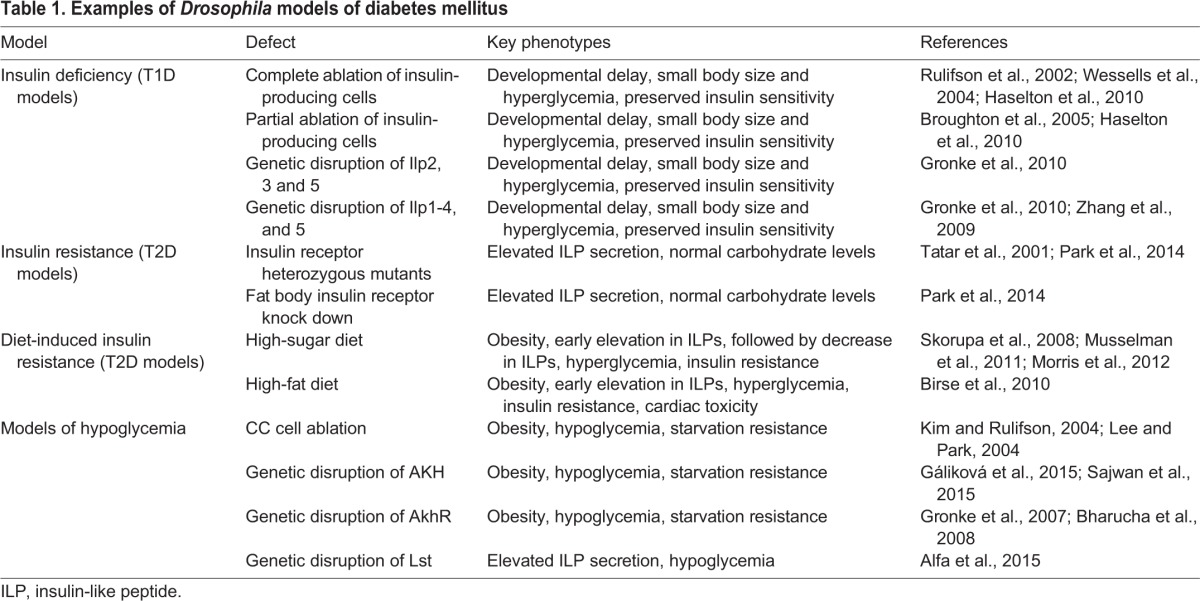
**Examples of *Drosophila* models of diabetes mellitus**

## Glucose homeostasis in *Drosophila*: a brief introduction

Circulating glucose levels in *Drosophila* are under the control of insulin-like peptides (ILPs) and the glucagon-like peptide adipokinetic hormone (AKH) (Ikeya et al., 2002; Kim and Rulifson, 2004; Lee and Park, 2004; Rulifson et al., 2002). Insulin-producing cells (IPCs) in adult flies synthesize three ILPs (Ilp2, Ilp3 and Ilp5; larval IPCs also produce Ilp1), and ablation of the IPCs or genetic deletion of Ilp2 causes hyperglycemia (Grönke et al., 2010; Haselton et al., 2010; Ikeya et al., 2002; Rulifson et al., 2002). The *Drosophila* fat body carries out metabolic functions performed by the mammalian adipose tissue and liver, including the storage and mobilization of energy reserves such as glycogen and fat (Ugur et al., 2016; Arrese and Soulages, 2010). As in mammals, insulin signaling in flies is a principal regulator of lipid accumulation (DiAngelo and Birnbaum, 2009). Lipid mobilization from the fat body is mediated by AKH and possibly by other hormones. AKH is produced by gut-associated endocrine cells called corpora cardiac (CC) cells. Mutation of the *Akh* gene or the gene encoding its receptor (*AkhR*), or the ablation of CC cells, result in severe obesity, hypoglycemia, and in lipid mobilization defects (Gáliková et al., 2015; Grönke et al., 2007; Kim and Rulifson, 2004; Lee and Park, 2004; Sajwan et al., 2015). Similar to glucagon signaling in mammals, AKH activates lipolysis through AkhR and through the fat body cAMP-dependent protein kinase A (PKA), via downstream mechanisms, many of which are as yet not fully understood (Arrese and Soulages, 2010; Bharucha et al., 2008; Patel et al., 2006; Staubli et al., 2002). Through tissue-specific manipulation of the IPCs and the fat body (and to a lesser degree the CC cells), investigators have thus far generated *Drosophila* models of both insulin deficiency and insulin resistance (described below, Table 1).

## Pathways that regulate insulin output

**Fig. 2.**
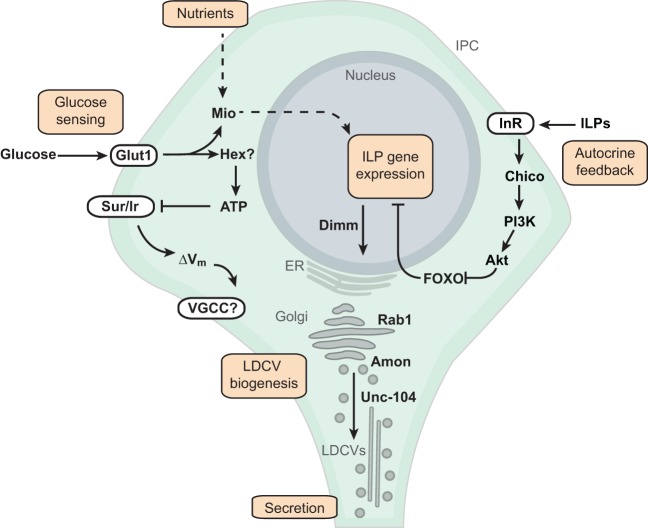
**Intrinsic regulators of insulin-like peptide output in *Drosophila*.** Schematic of a *Drosophila* insulin-producing cell (IPC) cell body and pathways involved in insulin-like peptide (ILP) production and secretion, including: transcription, translation, processing and secretion of ILPs. Dietary nutrients such as protein or carbohydrates control transcription of ILPs through unknown mechanisms, which might involve the glucose-responsive transcription factor Mio or IIS feedback signaling through FOXO. ILP expression also seems to be under autocrine control through insulin/IGF-like signaling (IIS). In response to IIS, FOXO is phosphorylated and retained in the cytoplasm, unable to activate expression of ILPs. A number of genes are important for the processing and packaging of ILPs into large dense core vesicles (LDVCs), including: Dimmed (Dimm), Rab1 GTPase, Amontillado (amon) and Unc-104 ortholog (Unc-104). In stimulus-secretion coupling, glucose enters the cell through Glut1 and is acted on by an unknown hexokinase (‘Hex?’) to generate ATP. ATP binds to the K_ATP_-channel subunit Sur and depolarizes the membrane (ΔV_m_) by decreasing conductance through an inward rectifying potassium channel (Ir). Fusion of LDCVs and insulin secretion occurs through activation of unknown voltage-gated calcium channels (VGCCs). See main text for details. ER, endoplasmic reticulum. Limited data exists for pathways indicated by hatched lines.

**Fig. 3.**
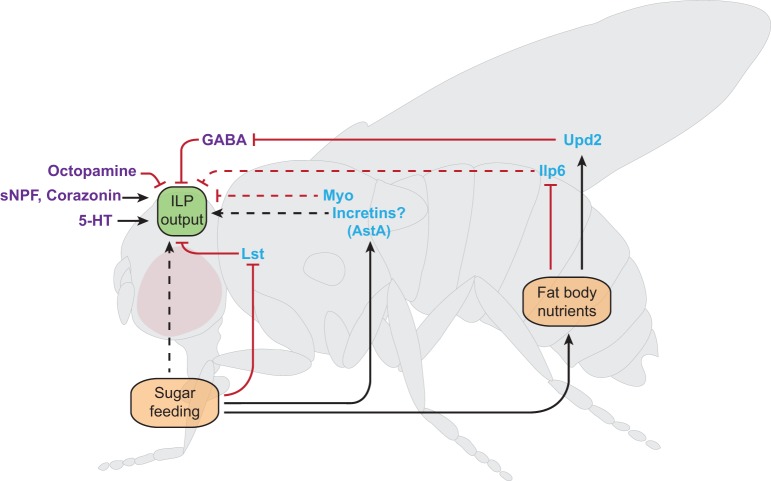
**Extrinsic regulators of insulin-like peptide output in *Drosophila*.** Schematic of an adult *Drosophila*, depicting extrinsic pathways that regulate insulin-like peptide (ILP) output from insulin-producing cells (IPCs). The location of IPCs in the central brain is indicated by the green box (ILP output). Neuromodulators and neuropeptide systems that modulate ILP secretion in the central brain are indicated in magenta text. Peripheral modulators of ILP secretion are indicated in blue text. Dietary sugar controls ILP output through the CC-cell-derived decretin hormone Limostatin (Lst), the fat body nutrient sensor and through unknown direct (hatched black lines) and incretin-like mechanisms. The fat body acts as a nutrient sensor to remotely control ILP output through secreted factors Unpaired2 (Upd2) and Ilp6. The myokinin Myoglianin (Myo) is secreted from muscle tissue and mediates inhibitory control over ILP output. sNPF, short Neuropeptide F; 5-HT, serotonin neurons; AstA, Allatostatin A. Positive pathways are shown by black arrows; inhibitory pathways are shown by red lines. Limited data exists for pathways indicated by hatched lines. See main text for additional discussion.

Insulin output reflects both insulin production and insulin secretion. Total insulin produced (insulin production) is the intracellular quantity of peptide available for secretion as a consequence of transcription, translation and post-translational processes, such as the biogenesis of large dense-core vesicles (LDCVs; Box 1) (Park et al., 2014). In contrast, secreted insulin (insulin secretion) refers to the quantity of peptide released into the circulation as a consequence of the cellular coupling of insulin secretion to circulating glucose (stimulus-secretion coupling; Box 1). Output can therefore be increased through increases in both production and secretion, or through increased secretion alone (Park et al., 2014). In *Drosophila*, insulin production and secretion can be assessed by measuring total ILP content in a single fly or by measuring circulating hemolymph ILP levels (Park et al., 2014). Below, we review mechanisms governing ILP output from the IPCs in *Drosophila*, beginning first with IPC-intrinsic processes regulating production and secretion (Fig. 2) and subsequently reviewing extrinsic neuromodulators and feedback circuits that modify ILP output in specific contexts (Fig. 3).

### Intrinsic IPC pathways that regulate insulin production

Although much is known about insulin transcription in mammals (Melloul et al., 2002), few studies have examined the transcriptional regulation of ILPs in flies: instead, much of the available data from flies reflects pathological or experimental states of insulin deficiency or studies of nutrient-dependent ILP synthesis. The IPC-derived ILP mRNAs are thought to be independently transcribed from genes located along a single *Drosophila* gene cluster on chromosome 3L (Grönke et al., 2010) and might be independently secreted (Kim and Neufeld, 2015). Genetic deficiency or loss of *Ilp2*, *Ilp3* or *Ilp5* increases transcription of the remaining ILPs (Broughton et al., 2008; Grönke et al., 2010), but whether this feedback is due to cell-autonomous mechanisms or homeostatic feedback regulation secondary to organismal insulin deficiency is not known. The forkhead transcription factor FOXO acts downstream of insulin/IGF-like signaling (IIS) as one mediator of insulin-dependent transcriptional activity in *Drosophila* (Puig and Tjian, 2005). IIS decreases nuclear occupancy of FOXO and, under conditions of low insulin, FOXO increases insulin sensitivity by directly stimulating transcription of the insulin receptor (InR) (Puig and Tjian, 2005). Whether IIS in the IPCs regulates ILP production in an autocrine manner is somewhat uncertain. ILP production has been shown to decrease upon activation of FOXO (Luong et al., 2006) but increase with expression of a dominant-negative InR (Broughton et al., 2005). Additional results suggest that reduction of InR in the IPCs decreases ILP secretion (Park et al., 2014), similar to results in mice (Kulkarni et al., 1999). The use of conditional expression systems to modulate IIS in adult IPCs could help to develop these findings.

Insulin transcription in *Drosophila* has also been studied in the context of nutritional status. Expression of *Ilp3* and *Ilp5*, but not *Ilp2*, is decreased with nutrient deprivation (Ikeya et al., 2002)*.* High-sugar or -protein feeding increases the expression of all three ILPs, but the precise mechanisms involved are not known (Buch et al., 2008; Kim and Neufeld, 2015; Musselman et al., 2011). The glucose-sensing transcription factor Mlx interactor (Mio) is an ortholog of the mammalian factor carbohydrate response element binding protein (ChREBP) and is expressed in IPCs. Although Mio is an appealing candidate for coordinating ILP expression and nutritional status, only Ilp3 is affected by Mio knockdown in *Drosophila* IPCs (Docherty et al., 2015). An important challenge in understanding transcriptional regulation of ILPs is that manipulations that cause ILP deficiency result in organism-wide defects in metabolic homeostasis, likely mobilizing multiple compensatory pathways.

Several human diabetes GWAS candidates encode transcription factors whose functions in regulating insulin transcription are currently unknown (Dimas et al., 2014). We have recently shown that knockdown of *lame duck* (*lmd*), a fly ortholog of the mammalian gene *GLIS3* (Yang et al., 2009), in the IPCs results in reduced ILP production (Park et al., 2014). Prior studies have associated the *GLIS3* locus both to T1D and T2D susceptibility in humans (Barrett et al., 2009; Dupuis et al., 2010; Nogueira et al., 2013). In the case of *lmd* loss of function in flies, we observed a decrease in *Ilp2* mRNA, and consequently both total and circulating protein levels are decreased, emphasizing the requirement for ILP transcription in maintaining normal ILP output (Park et al., 2014). Alternatively, transcription factors regulating expression of genes encoding factors required for insulin processing or IPC secretory components would be expected to produce limited defects in production or secretion, respectively. Leveraging the ease of genetic screens in the fly and a focused set of robust assays, investigators are able to quickly interrogate scores of transcription factors to validate GWAS candidates or identify networks involved in insulin production or secretion.

In flies and mammals alike, insulins are translated as precursor pro-peptides (pre-propeptides; Box 1) that are packaged into secretory granules, where they undergo post-translational processing prior to secretion. Similar to pancreatic β-cells, *Drosophila* IPCs produce LDCVs (Cao et al., 2014; Hadžić et al., 2015). Genes that modulate post-translational processing and the biogenesis of LDCVs also affect ILP production (Fig. 2). Although flies lack a known ortholog of the mammalian insulin-processing enzyme prohormone convertase 1 (PC1; Box 1), a *Drosophila* homolog of PC2 called Amontillado (Amon) is expressed in the IPCs (Rayburn et al., 2009). Mutants for *amon* exhibit ILP deficiency phenotypes, supporting a role for Amon in the processing of ILPs (Rayburn et al., 2009). In rodents, the dominant ‘*Akita*’ allele encodes an insulin precursor with conformational/processing defects that is retained in the endoplasmic reticulum (ER), leading to ER stress, decreased insulin production, loss of β-cells and T2D phenotypes (Ron, 2002; Wang et al., 1999). Similarly, flies harboring an orthologous ‘*Akita*’ allele of *Ilp2* exhibit phenotypes of ILP deficiency, suggesting parallels in the early post-translational processing of insulins among flies and mice (Park et al., 2014).

The Rab family of GTP-binding proteins is important in trafficking and sorting of LDCVs in mammals (Suckale and Solimena, 2010). In *Drosophila*, Rab1 along with the kinesin Unc-104 have been shown to be crucial for ILP production and axonal transport in the IPCs (Cao et al., 2014). The transcription factor Dimmed (Dimm) also regulates LDCVs in *Drosophila* through transcriptional regulation of a large number of genes required for LDCV assembly (Hadžić et al., 2015). Thus, Dimm might function as an effector for scaling ILP production by allowing the cell to quantitatively increase its secretory capacity in response to physiological demands (Mills and Taghert, 2012). Thus, Dimm might serve as a mediator for scaling ILP production, and defects in this pathway might underlie some states of failed IPC compensation when challenged by insulin resistance.

### Intrinsic IPC pathways that regulate insulin secretion

In mammals, insulin secretion is tightly coupled to serum glucose levels through stimulus-secretion coupling, which begins with the transduction of intracellular ATP to membrane depolarization (Fig. 2; Box 1). Glucose is taken up by human β-cells through the GLUT1 transporter and is processed by the glycolytic enzyme glucokinase, eventually generating ATP, which inactivates ATP-gated potassium (K_ATP_) channels to depolarize the β-cell membrane (ΔV_m_). Mutations in glucokinase (*GCK*) or genes encoding the K_ATP_-channel subunits result in heritable forms of diabetes (Ashcroft and Gribble, 1999; MacDonald et al., 2005). An ortholog of the ATP-sensing subunit of the K_ATP_ channel, encoded by *Sur*, is present in *Drosophila*. In larvae, Sur is expressed in the CC cells but not the IPCs, and the latter seem to lack intrinsic glucose-sensing at this stage (Kim and Rulifson, 2004; Nässel et al., 2015). Instead, larval IPC secretion might be regulated through nutrient sensing and signaling from larval fat-body adipose tissue (Géminard et al., 2009). However, in adults, K_ATP_-channel activity and glucose-dependent excitation are present in the IPCs (Kréneisz et al., 2010). ILPs are secreted following an oral glucose challenge in adult flies, and ILP secretion in this context is abrogated by loss of the membrane glucose transporter Glut1 (Park et al., 2014). In summary, IPCs seem to lack glucose-sensing machinery in the larvae and likely develop this functional capacity during or after metamorphosis. In mammals, glucose-sensing in pancreatic β-cells is acquired shortly after birth through a poorly understood process referred to as β-cell maturation (Aguayo-Mazzucato et al., 2011; Avrahami et al., 2015). β-cell maturation is an important limiting step for generating functional β-cells – which could be used to replenish those that are lost in T1D – from renewable tissue sources, and intensive world-wide efforts are focused on advancing this area of β-cell biology (Blum et al., 2012). An understanding of the genes and developmental processes involved in maturation of larval IPCs into their adult, glucose-sensing counterparts in *Drosophila* could advance research into this area.

The closure of the K_ATP_ channels in mammalian β-cells results in depolarization and in the activation of voltage-gated calcium channels (VGCCs) and sodium-conductance channels (Rorsman and Braun, 2013). By comparison, the corresponding channel repertoire and electrophysiology underlying *Drosophila* IPC function remains largely unknown. Levitan and colleagues recently identified a role for the *Drosophila* calcium- and voltage-sensitive potassium (BK) channel, Slowpoke (Slo), in regulating *in vivo* action-potential duration in neurons in the anterior midline [the pars intercerebralis (PI)] (Shahidullah et al., 2009). They show that mutations in a negative regulator of Slo, Slo-binding protein (Slob), produce hypoglycemia and elevations in Akt phosphorylation consistent with increased secretion of ILPs (Sheldon et al., 2011). In murine pancreatic β-cells, loss of BK channels similarly increases action-potential duration, resulting in insulin secretion defects (Düfer et al., 2011). Although a murine or human homolog of Slob has not yet been identified, results from the fly suggest that regulators of BK channels might be important in modulating insulin secretion in mammals (Sheldon et al., 2011).

Over the past decade, technical advances have permitted investigators to perform *in vivo* cellular and ion-channel physiology in flies (Fridell et al., 2009; Kréneisz et al., 2010; Shahidullah et al., 2009; Tian et al., 2009). Using these methods, investigators can now begin to probe the *in vivo* physiology of IPC activity and glucose homeostasis in the fly. For example, although glucose sensing in cultured IPCs has been demonstrated (Kréneisz et al., 2010), *in vivo* glucose sensing has not yet been demonstrated in the adult fly. Using *in vivo* calcium imaging in flies, investigators can address questions such as whether IPCs respond differently to oral sugar stimuli in comparison to changes in hemolymph sugar levels (see information on incretins below). Importantly, the use of cellular and ion-channel-physiology methods permit more nuanced dissection of the mechanisms underlying molecular interventions. For example, we recently identified the transcription factor *CG9650* as an IPC-intrinsic regulator of ILP secretion but not of ILP production (Park et al., 2014). *CG9650* encodes an ortholog of the human zinc-finger transcription factor *BCL11A*, previously linked by GWAS to T2D risk in humans (Wheeler and Barroso, 2011). We speculate that *CG9650* effects on ILP secretion might reflect regulation of glucose-dependent activity, vesicle trafficking or vesicle fusion of IPCs (Sangbin Park and S.K.K., unpublished results). In summary, advances in our ability to measure adult fly IPC physiology and ILP secretion should enable functional studies of postulated IPC regulators such as *CG9650*.

### Extrinsic pathways that regulate insulin production and secretion

In both mammals and flies, secretion of insulins is highly influenced by secreted factors and signals external to the insulin-secreting endocrine cells (Nässel et al., 2013; Rorsman and Braun, 2013). We refer to two types of extrinsic regulators in the sections that follow: (1) the modulation of IPCs by neurons in the central brain, and (2) the hormonal modulation of IPCs by remote endocrine and peripheral tissues (Fig. 3). These pathways affect insulin production, secretion, or both. In modulating insulin output, extrinsic pathways permit the coupling of IIS to organismal and nutritional states, as will be described in detail below.

*Drosophila* IPCs are located in the PI and are under the direct control of neuromodulatory neurons and neurotransmitters (reviewed in Nässel et al., 2013). Serotonin [5-hydroxytryptamine (5-HT)] neurons were among the first to be implicated in controlling ILP secretion: Scott and colleagues found that loss of the nucleostemin family GTPase NS3 in these cells produced ILP deficiency phenotypes (Kaplan et al., 2008). Follow-up studies identified the inhibitory 5-HT_1A_ receptor as a target for 5-HT signaling in IPCs and showed that a reduction in the levels of this receptor increased ILP transcription (Luo et al., 2012). Thus, 5-HT might be an inhibitor of ILP production (Nässel et al., 2013). Octopamine, an insect functional analog of norepinephrine (noradrenaline), also modulates the activity of IPCs and controls sleep and wake cycles via the Octopamine receptor (OAMB) (Crocker et al., 2010). However, reduction of OAMB in IPCs decreases Ilp3 expression, but has no effect on carbohydrate metabolism (Luo et al., 2014). The inhibitory amino acid neurotransmitter GABA has also been implicated in the control of insulin production by IPCs. Initial studies suggested that central GABAergic neurons adjacent to the IPCs provide inhibitory regulation of the IPCs via the GABA_B_ receptor (Enell et al., 2010). Subsequent studies suggest that GABAergic inhibition of the IPCs is modulated by nutrition-dependent signaling from the fat body via the leptin-like hormone Unpaired 2 (Upd2) (Rajan and Perrimon, 2012). GABAergic input provides continuous inhibition of IPCs that is lifted through the inhibition of these neurons by Upd2 (Rajan and Perrimon, 2012). Finally, evidence supports the role of the peptide hormones short neuropeptide F (sNPF) and corazonin in modulating ILP production. Reduction of these neuropeptides in the *Drosophila* brain results in hyperglycemia, which is thought to reflect ILP deficiency (Kapan et al., 2012; Lee et al., 2009; Nässel et al., 2013). Thus, multiple neuronal signaling systems have been implicated as regulators of ILP secretion in the fly (Fig. 3), and most of these have mammalian counterparts. Future studies of these systems should help to decipher physiological, behavioral or pathological settings in which these systems modulate IPC activity or function.

Although insulin secretion is principally coupled to circulating glucose levels in mammals, glucose homeostasis also requires the pancreatic β-cell to integrate a large number of endocrine signals secreted from tissues outside the pancreas. The *Drosophila* fat body was among the first tissues discovered to exert ‘remote control’ over the insulin-producing cells (Géminard et al., 2009). Upd2 (discussed above) signals the fed state to IPCs, promoting the release of insulin (Rajan and Perrimon, 2012). However, the fat body also inhibits IPCs under conditions of fasting. This effect seems to be mediated by another insulin-like peptide, Ilp6, levels of which are increased during fasting (Bai et al., 2012). Notably, Ilp6 expression is increased by the activation of FOXO signaling under conditions of starvation or low IIS in the fat body (Bai et al., 2012; Hwangbo et al., 2004). Similarly, FOXO signaling in the flight muscles reduces ILP levels in the IPCs, and this effect is also mediated by a secreted signal, the *Drosophila* myokinin Myoglianin (Demontis and Perrimon, 2010; Demontis et al., 2014). Lastly, results suggest that the *Drosophila* adiponectin receptor AdipoR stimulates insulin secretion from IPCs, although a fly adiponectin remains to be identified (Kwak et al., 2013). In summary, through a diverse set of secreted signals, the fat body performs an important function in modulating glucose homeostasis by signaling ambient organismal nutrient status to the IPCs. Similarly, in mammals, hormone signals from the liver, such as kisspeptin, are thought to regulate insulin output (Song et al., 2014).

In mammals, the gut also modulates insulin secretion during feeding through incretin hormones produced by enteroendocrine cells (Baggio and Drucker, 2007; Campbell and Drucker, 2013; Gribble and Reimann, 2016). Incretins do not stimulate insulin secretion directly; rather, they amplify glucose-stimulated insulin secretion (Campbell and Drucker, 2013). Gut-derived hormones that actively suppress insulin secretion after starvation in mammals – decretins – had been postulated but not identified (Unger et al., 1963). *Drosophila* Limostatin (Lst) was recently identified as the first decretin (Alfa et al., 2015). Levels of Lst are increased during fasting in gut-associated CC cells and this suppresses ILP production and secretion through the G-protein-coupled receptor encoded by *CG9918* (Alfa et al., 2015)*.* Neuromedin U receptor 1 (*NMUR1*) is a mammalian ortholog of *CG9918*, and is expressed in human pancreatic β-cells, whereas its cognate ligand, NMU, is produced in enteroendocrine cells of the stomach and intestines (as well as in brain neurons), and suppresses glucose-stimulated insulin secretion by human islets (Alfa et al., 2015). Decretins might therefore represent an ancient and conserved hormone class for attenuating insulin responses when nutrients are scarce. We speculate that decretins might help to sustain circulating post-prandial glucose levels in this setting, thereby preventing neuroglycopenia that might follow limited refeeding. Based on these findings, we also postulate that additional entero-insular hormones in the fly, including incretins, remain to be discovered. Both the CC cells and enteroendocrine cells lining the *Drosophila* gut produce a large number of secreted peptides that remain incompletely characterized (Baggerman et al., 2002; Predel et al., 2004). For example, recent findings suggest that *AstA* mRNA increases in *Drosophila* enteroendocrine cells after feeding, especially after carbohydrate feeding, and that AstA can signal to IPCs and CC cells (Hentze et al., 2015). It remains to be determined whether AstA or other hormones potentiate ILP secretion and therefore perform incretin-like functions in flies.

It is apparent from results in both *Drosophila* and mammals that the regulation of circulating insulin levels is complex, involving the convergence of signals from many tissues onto the IPCs. Disruption of genes involved in intrinsic pathways of insulin production tends to produce developmental and metabolic phenotypes that reflect prolonged ILP deficiency. In contrast, disruption of extrinsic pathways often produces more subtle, metabolically restricted phenotypes without developmental delay or changes in size, and might be masked by compensation (Park et al., 2014). Undoubtedly, further studies will show that extrinsic regulators regulate ILP output by converging on the function of intrinsic components. At a ‘systems physiology’ level, regulation of fly hormones such as ILPs and Lst by behavior and metabolism might serve as a crucial link between insulin production and insulin resistance (discussed below).

## Pathways that regulate insulin sensitivity

**Fig. 4.**
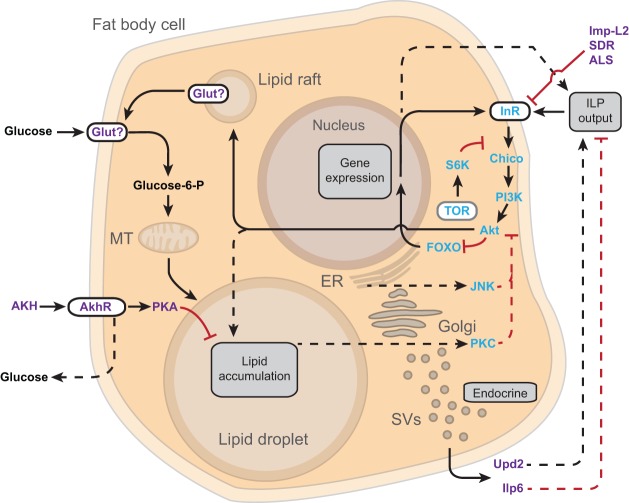
**Insulin resistance and feedback in the *Drosophila* fat body.** Schematic depiction of a *Drosophila* fat body cell illustrating gene products that might contribute to primary (blue text) and secondary (magenta text) mechanisms of insulin resistance. Primary mechanisms: alterations of insulin receptor (InR) or any downstream components of the insulin/IGF-like signaling (IIS) pathway lead to insulin resistance. Target of rapamycin (TOR) and Jun-N-terminal kinase (JNK) pathways are proposed to negatively regulate IIS signaling and thus contribute to insulin resistance. Lipid accumulation [which occurs when flies are fed a high-sugar diet (HSD); see main text] leads to PKC activation, which contributes to insulin resistance through negative feedback on IIS. Secondary mechanisms: the secreted insulin-binding proteins Imp-L2, Secreted decoy of InR (SDR) and Acid-labile subunit (ALS) cause insulin resistance by interfering with the binding of insulin to its receptor. IIS triggers membrane localization of an unknown *Drosophila* glucose transporter (Glut?) through membrane fusion of lipid rafts to facilitate removal of hemolymph glucose, and defects in this glucose transporter or its trafficking might lead to insulin resistance. AKH stimulates lipolysis (involving AkhR and PKA), and defects in this pathway lead to obesity. AKH also increases hemolymph glucose through unknown mechanisms that likely involve gluconeogenesis and breakdown of glycogen, and might contribute to hyperglycemia in diabetic states. Fat body secreted factors (endocrine, lower right) Unpaired2 (Upd2) and insulin-like peptide 6 (Ilp6) might contribute to insulin resistance in certain nutritional states. PKA, Protein kinase A; AKH, Adipokinetic hormone; AkhR, Adipokinetic hormone receptor; ER, endoplasmic reticulum; SVs, secretory vesicles; MT, mitochondrion. Positive pathways are shown by black arrows; inhibitory pathways are shown by red lines. Limited data exists for pathways indicated by hatched lines. See main text for additional discussion.

Insulin resistance is another major pathophysiological mechanism that underlies glucose intolerance and T2D in mammals (Eckel et al., 2011; Samuel and Shulman, 2012). In the fed state, circulating carbohydrates are plentiful and the anabolic actions of insulin predominate, including glucose uptake by the liver in mammals (and by the fat body in *Drosophila*), as well as glycogen synthesis and decreased lipolysis (Samuel and Shulman, 2012). Under conditions of insulin resistance, peripheral tissues fail to respond to insulin, resulting in hyperglycemia, dysregulated glycogen synthesis and elevation of circulating free fatty acids from inappropriate lipolysis (Samuel and Shulman, 2012). In considering the genetic contributors to insulin resistance, we refer to primary mechanisms as genetic defects that affect IIS directly and secondary mechanisms as genetic defects that contribute to insulin resistance phenotypes but do not affect IIS directly (Fig. 4). Below, we review important areas of investigation into the mechanisms of insulin resistance and provide relevant examples for each of these mechanisms in flies.

### Primary mechanisms of insulin resistance

*Drosophila* that are heterozygous for the mutant InR allele *InR^05545^* (*InR^05545^/InR^+^*) exhibit reduced InR activity but otherwise have normal circulating carbohydrates (Park et al., 2014; Tatar et al., 2001). Although ILP production is unchanged in these flies, circulating ILP levels are elevated, indicating an isolated increase in ILP secretion. Targeted reduction of *InR* transcript in the fat body alone is sufficient to recapitulate these phenotypes, supporting the primacy of fat body IIS in this phenotype (Park et al., 2014). The fat body in *Drosophila* performs functions of both adipose and liver in mammals, and these experiments closely mirror findings from liver-specific *InR*-knockout (LIRKO) mice (Michael et al., 2000). However, whether compensatory ILP secretion in this context is mediated by a fat-body-derived signal [e.g. Upd2, Ilp6 (discussed above)] or an increase in glucose-stimulated secretion from the IPCs secondary to the reduction in glucose disposal by the fat body remains unknown.

Insulin-resistant *Drosophila* have also been generated by rearing flies on high-sugar diet (HSD) (Morris et al., 2012; Musselman et al., 2011; Skorupa et al., 2008) or high-fat diet (HFD) (Birse et al., 2010). Similar to *InR^05545^/InR^+^* insulin-resistant flies, HSD causes insulin resistance with ILP compensation. However, after sustained HSD, ILP expression decreases and these flies develop hyperglycemia (Morris et al., 2012; Musselman et al., 2011). Again, it is not known whether fat body signals in this model provide feedback signaling to the IPCs. We have shown that high-sugar feeding produces a robust suppression of Lst, a negative regulator of ILP production and secretion (Alfa et al., 2015). Thus, it is possible that reduction of Lst serves as one mechanism for increased ILP output in this model. An important distinction between the HSD and *InR^05545^/InR^+^* insulin-resistance models is that, in the latter, compensation remains appropriate, whereas, with HSD, compensation fails and these flies become hyperglycemic. The reasons for this are not known, but could be related to the increase in lipid accumulation in HSD flies (see below), and might be relevant to mammalian T2D pathogenesis. Mice challenged with HFD initially show β-cell compensation, with relative hyperinsulinemia and normoglycemia, but this is invariably followed by β-cell failure, impaired insulin secretion and hyperglycemia (Kasuga, 2006). Although ‘lipotoxicity’ has been invoked as one reason for this ‘β-cell failure’ during HFD challenge, the molecular mechanisms of islet β-cell failure remain incompletely understood (Samuel and Shulman, 2012). Thus, ‘IPC failure’ evoked by nutrient challenge in flies might be useful for understanding conserved facultative or maladaptive responses by IPCs.

Lipid accumulation in peripheral tissues, including in the liver and adipocytes, might itself be a causative factor in insulin resistance, although the precise mechanisms involved remain difficult to establish (Fabbrini et al., 2009; Krssak et al., 1999; Samuel and Shulman, 2012). In flies, HSD models lead to elevated ILP levels and lipid accumulation in the fat body (Morris et al., 2012; Musselman et al., 2011). Notably, elevated IIS in the fat body alone is sufficient for lipid accumulation (DiAngelo and Birnbaum, 2009). In mammals, lipid accumulation causes activation of protein kinase C (PKC) in adipocytes, which has been linked to insulin resistance (Samuel and Shulman, 2012). *Drosophila* (S2) cell experiments have also shown that activated PKC antagonizes insulin signaling (Mattila et al., 2008). These results support a model whereby elevated ILP levels in HSD stimulates lipid accumulation, which inhibits fat body IIS, causing insulin resistance through activation of PKC. Interestingly, adiposity and lipid accumulation might also protect against insulin resistance and hyperglycemia in some contexts. In mammals, the Tubby gene (*Tub*) belongs to a family of genes of unknown function and mutation of this gene has been found to cause obesity in rodents, with persistent elevation of insulin, but without development of diabetes (Coleman and Eicher, 1990). Flies deficient for the *Drosophila* ortholog of Tubby (King tubby) and reared on HSD are obese but protected against hyperglycemia (Musselman et al., 2013). Lipid accumulation contributes to insulin resistance by inhibiting IIS, but results from Tubby experiments suggest that this pathway might become active only after the full capacity for lipid accumulation has been reached.

An extensive discussion of intracellular signaling pathways is beyond the scope of this Review; however, the role of IIS components and their interaction with tuberous sclerosis complex (TSC1-2)/target of rapamycin (TOR) and of Jun-N-terminal kinase (JNK) pathways in insulin resistance are important areas for ongoing investigation (Chantranupong et al., 2015; Oldham, 2011; Samuel and Shulman, 2012; Shah and Hunter, 2014). *InR* is itself a transcriptional target of FOXO, and excess insulin signaling exerts negative feedback that decreases the production of InR protein as well as its key downstream mediator, insulin receptor substrate (IRS) (Evans et al., 2011; Puig and Tjian, 2005). Hence, states of nutritional excess and overactive InR signaling dampen the responsiveness of peripheral tissues to insulin ligand (Marr et al., 2007; Puig and Tjian, 2005). FOXO is activated upon low IIS conditions and has been shown to sensitize insulin responses in *Drosophila* and mammals (Matsumoto et al., 2006; Puig and Tjian, 2005). Consistently, constitutive activation of FOXO leads to lipid accumulation in the fat body of flies, as well as suppression of *Ilp* mRNA (Hwangbo et al., 2004; Luong et al., 2006). TOR is an ancient and highly conserved nutrient-sensing pathway that is sensitive to amino acids (Chantranupong et al., 2015), and reduction of TOR activity results in starvation phenotypes (Oldham et al., 2000). Conversely, activation of TOR complex 1 (TORC1) components stimulate insulin resistance in mammalian cells (Shah et al., 2004). The TORC1 effector S6K1 exerts negative feedback on IIS (Kockel et al., 2010) and loss of this effector is protective against HFD-induced insulin resistance in mice (Um et al., 2004). Thus, TOR signaling is likely an important mediator of insulin resistance during nutritional excess.

In mammals, activation of JNK signaling has also been linked to insulin resistance, and reduction of JNK signaling has been shown to be protective against diet-induced insulin resistance (Hirosumi et al., 2002; Samuel and Shulman, 2012). Studies from *Drosophila* have shown that peripheral JNK signaling might antagonize IIS through activation of FOXO or by the secretion of IIS inhibitory factors (Biteau et al., 2011; Wang et al., 2005). Given that activation of FOXO increases *InR* expression and insulin sensitivity (Puig and Tjian, 2005), it is possible that insulin resistance upon JNK activation involves additional or context-dependent mechanisms. Consistent with the role of IIS inhibitors in JNK-dependent insulin resistance, reduction of the secreted *Drosophila* JNK target Neural lazarillo (NLaz) is protective against HSD-induced insulin resistance (Pasco and Léopold, 2012). In summary, although there is accumulating evidence to support the role of TOR and JNK signaling in contributing to insulin resistance (Fig. 4), the complex interactions of these pathways and IIS leave much to be revealed. Future studies that combine HSD with tissue-specific gene manipulation in *Drosophila* will undoubtedly contribute additional insights.

### Secondary mechanisms of insulin resistance

GLUT4 is the major mammalian insulin-responsive glucose transporter involved in glucose uptake by adipose and muscle cells (glucose disposal), and loss of GLUT4 in mice results in insulin resistance (Stenbit et al., 1997). Unlike primary insulin signaling defects (described above), insulin resistance in these mice constitutes an inadequate response to insulin due to a defect in a target of the pathway. Although an orthologous *Drosophila* glucose transporter in the fat body has not been characterized, exogenous human GLUT4 impacts the responses to insulin in the *Drosophila* fat body (Crivat et al., 2013). These results suggest conserved mechanisms of insulin-dependent glucose transport in *Drosophila*, and support the possibility of identifying uncharacterized components of these pathways as putative diabetes susceptibility genes.

Insulin resistance in the context of preserved target-cell signaling and intracellular pathways can also be induced by interference with the binding of insulin to InR. One of the earliest reports of such a mechanism described a rare form of insulin resistance in humans caused by InR auto-antibodies, which prevented the binding of insulin to its receptor (Flier et al., 1976). Recent studies have identified insulin-like growth factor binding proteins (IGFBPs) as contributors to insulin resistance in mammals. A screen for negative regulators of IIS in *Drosophila* identified the IGFBP7 ortholog Imp-L2 (Honegger et al., 2008). Interestingly, Imp-L2 is secreted by *Drosophila* tumors and might underlie insulin resistance and organ wasting in malignant states (Figueroa-Clarevega and Bilder, 2015; Kwon et al., 2015). Notably, elevated serum levels of IGFBP7 are associated with insulin resistance and metabolic syndrome in humans (Liu et al., 2015). Two additional ILP-binding hemolymph proteins, Secreted decoy of InR (SDR) and Acid-labile subunit (ALS), have been described in *Drosophila* and might contribute to insulin resistance by similar mechanisms as described above (Arquier et al., 2008; Okamoto et al., 2013).

Glucagon excess and the accompanying increase in hepatic glucose production are associated with insulin-resistant diabetic states in mammals (Brown et al., 2008; Shah et al., 2000; Unger and Orci, 2010). Like glucagon, *Drosophila* AKH increases circulating glucose and stimulates lipolysis (Braco et al., 2012; Kim and Rulifson, 2004). However, it is not known whether AKH contributes to the hyperglycemia in insulin resistance or insulin deficiency*.* Increased transcription of *AkhR*, but not of *AKH*, is observed in HSD but this does not establish increased AKH activity (Musselman et al., 2011). Increased AkhR expression could reflect compensation for decreased AKH secretion in the context of persistent hyperglycemia (Alfa et al., 2015). Alternatively, insulin deficiency might stimulate AKH activity owing to insulin resistance in the CC cells. Although AKH peptide levels have not been measured directly, assessing the relative contribution of AKH to hyperglycemia in insulin-deficient states can be done genetically and remains an open question.

## Future directions

In the past decade, investigators have established *Drosophila* as a model organism for studying insulin signaling and metabolic pathways relevant to human diseases like T1D and T2D (see Table 1). Nonetheless, several areas provide opportunities for advancing the field of *Drosophila* metabolism and providing new insights into human metabolic disease: (1) the development of new methods, such as for measuring hormones in metabolic studies of feeding, fasting and obesity in *Drosophila*; (2) the *in vivo* characterization of human diabetes susceptibility genes and their mechanisms of function; (3) the establishment of integrative physiology studies across multiple organ systems and pathophysiological contexts in flies to understand how individual genes and tissues converge to maintain the homeostasis of glucose, lipids and other metabolites; and (4) studies of the evolution and development of glucose-responsive insulin output.

The heterogeneity of approaches used in metabolic studies presents an important challenge in synthesizing findings. For example, studies have been performed in both larval-staged flies and adult flies, and increasing evidence suggests that metabolic physiology differs between these stages (Alfa et al., 2015; Kim and Rulifson, 2004). Early studies used a variety of methods for assessing some of the same parameters (e.g. hemolymph glucose) and, previously, measurement of ILP levels was not possible. In the future we suspect that the use of standardized methods for metabolic assays (Tennessen et al., 2014), along with newly developed methods to quantify systemic levels of hormones like Ilp2 (Park et al., 2014), Akh and Lst, will improve the translation of findings from flies to mammalian biology.

With improved methods in hand, researchers can investigate the function of candidate human diabetes susceptibility genes and perhaps identify additional modulators of insulin biology and metabolism (Dimas et al., 2014; Pendse et al., 2013; Renström et al., 2009; Zhao et al., 2010). One study has already performed a large-scale phenotypic assessment of candidates in *Drosophila*, identifying a homolog of the human homeobox-domain transcription factor *HHEX* (Pendse et al., 2013). Similarly, we performed an initial screen of human GWAS-identified candidates using a combination of ILP ELISA methods and metabolic assays to identify specific roles for orthologs of *GLIS3* and *BCL11A* in regulating insulin production or insulin secretion by IPCs (Park et al., 2014). Using the combination of tissue-specific manipulations and HSD, it might be possible to assess the role of candidate genes in modifying susceptibility to insulin resistance or hyperglycemia. We also foresee that studies of insulin regulation in different *Drosophila* species, which have distinct phenotypes such as size, longevity and adiposity, might uncover evolutionarily-honed mechanisms for defining a ‘set-point’ for insulin secretion after feeding in different species.

Understanding the nature of gene-environment interactions in T2D susceptibility is an important goal (Bouret et al., 2015). Although GWAS candidates provide a critical starting point, much work remains in both identifying additional genetic risk factors for T2D and characterizing their functions. These efforts will undoubtedly be accelerated through the use of *Drosophila* models to combine genetics, physiology and dietary manipulations. In the past decade, the field of hormone biology, metabolism and diabetes research in *Drosophila* has identified parallels by drawing on the wellspring of knowledge about physiological and adaptive mechanisms of glucose and lipid homeostasis in mammals. In the coming decade, we anticipate a growing reversal of this information stream, where findings in *Drosophila* will increasingly presage discoveries about physiological homeostasis, hormone regulation and metabolism in mammals.
